# Mental health services and resources for children with developmental disabilities and their families: scan of local practices, gaps, and opportunities created

**DOI:** 10.3389/fresc.2023.1118769

**Published:** 2023-05-30

**Authors:** Jessica Hanson, Kayla Heslon, Tatiana Ogourtsova

**Affiliations:** ^1^ Integrated Center of Health and Social Services of Laval, The Research Center of the Jewish Rehabilitation Hospital, Laval, QC, Canada; ^2^ Faculty of Medicine and Health Sciences, School of Physical and Occupational Therapy, McGill University, Montreal, QC, Canada; ^3^ Centre for Interdisciplinary Research in Rehabilitation of Greater Montreal (CRIR), Montreal, QC, Canada; ^4^ Faculty of Arts and Sciences, McGill University, Montreal, QC, Canada

**Keywords:** mental health services, childhood disability, environmental scan, neurodevelopmental disability, COVID-19 pandemic

## Abstract

**Background:**

Mental health concerns in children with disabilities are common and have a significant and negative impact. Clinicians have reported high demand for this population to receive early, targeted, and family-centred mental health interventions.

**Objective:**

We sought to map out and describe existing pediatric mental health services/resources for children with disabilities and their families across clinical sites and local and online communities.

**Methods:**

Using a mixed-method triangulation study design, we outreached to clinical managers at the participating clinical sites and conducted a rapid online search of local in-person, telehealth, and web-based information. The nature, access method, admission criteria, target, focus, and other pertinent information were recorded and analyzed using descriptive statistics and a narrative synthesis approach.

**Results:**

Eighty-one (*n* = 81) services/resources (in-person, *n* = 48; telehealth, *n* = 10; web-based information, *n* = 33) were identified. Few (*n* = 6, 13%) in-person services had a method of care access through an online booking portal. Nearly half of in-person resources (*n* = 23, 47%) had admission criteria specific for children with disabilities (e.g., diagnosis, age limit), and many (*n* = 32, 67%) required a formal referral. A small number of in-person and telehealth services targeted the mental health concerns of the entire family (*n* = 23, 47%; *n* = 2, 20%). Very few (*n* = 13, 16%) services incorporated follow-up support. Important gaps emerged for certain populations (e.g., children with cerebral palsy). Practitioners’ inadequate training when intervening with co-existing mental health demands of children with disabilities was noted by clinical managers.

**Conclusion:**

Findings could be used to create a user-friendly database to easily identify suitable services and to advocate for services/resources that are lacking.

## 1. Introduction

One in eleven children between the ages of 4–11 lives with a neurodevelopmental disability (NDD, e.g., cerebral palsy, autism spectrum disorder), making up 9.2% of all children in Canada (1). NDD typically emerges during early childhood and results in impaired functioning (2, 3). Nearly every fifth child with an NDD presents a co-occurring mental health issue, such as anxiety and depression, that negatively impacts their daily functioning, participation, and quality of life (4, 5). Caregivers and clinicians have expressed challenges in accessing or utilizing available resources effectively to meet the mental health needs of children with NDD (6, 7). Canadian reports show there are many pre-existing issues in the availability, relevance, and quality of mental health services for children (8). Child mental health services are often characterized by prolonged waiting periods and inadequate accountability measures for allocating public funds (9). In addition, for children with NDD, individual, social, physical, organizational, and systemic barriers may further limit access to existing resources (10).

The COVID-19 pandemic has affected the mental health of all individuals worldwide (11). However, children with NDD and their parents faced (and continue to bear) an even more heightened psychological burden (12). With medical care being prioritized for those directly affected by the virus, along with the closure of mental health clinics, necessary mental health support has not been easily accessible for children with NDD and their families, serving as an additional source of stress, anxiety, and fear (13). A Canadian report states that many children and youth are waiting months and even years to access specialized mental health care and treatment, and it is imperative that the government commits to broader mental health system improvement (14). Moreover, for many children with NDD, maintaining a daily routine is essential for their psychological and emotional well-being (13). Adjustments to established daily schedules (e.g., school closures, cancelled after-school activities, frequent testing procedures, social distancing, and confinement) can be a real struggle for children with NDD (15).

Furthermore, shifting to online learning has led to a dearth of special education assistance that these children require (16). Coupled with this, being confined at home during the pandemic resulted in an elevated level of helplessness in children and parents alike (13). Caregivers are reported to face heightened feelings of worry and stress, given insufficient resources and inadequate knowledge on how to best support their child with special needs (17–19). Overall, the pandemic has exacerbated the mental health challenges for children with NDD and their parents, leading to an even greater demand for resources and services.

The province of Quebec (Canada) was “hit hard” during the pandemic, representing 22.57% of the national population, with >52% of confirmed cases and >64% of deaths (20). More than one-third of these confirmed cases were identified in Montreal (Quebec, Canada) (21). Like many other urban cities during the pandemic, Montreal faced several long-term school closures, leaving students learning online at home for months at a time (22). Fifty-six percent of Montreal caregivers say that the isolation and the loneliness their child inherently faced during school closures has deteriorated their child's mental health (22). Additionally, parents of children with disabilities were left stressed and exhausted, with no support to meet their child's special care and education needs at home (23). As 35.8% of Canada's low-income neighbourhoods are in Montréal, many families cannot afford access to private mental health services that may be more accessible (24). In socialized medical settings in Montreal, clinicians have reported a significant rise in the number of NDD patients with emerging mental health concerns. Additionally, they state that existing public mental health services are neither standardized nor nimble enough to adequately address and support the mental health demands of children with NDD (25, 26).

As a first step to improving the quality of mental health services for this population, we sought to construct a consolidated contextual map of existing mental health services/care pathways for children with NDD and their families. The purpose of this brief report was to provide key stakeholders (i.e., clinicians, decision-makers/clinical coordinators, patients, and families) with knowledge about the current position of the available services and present an experience of mapping the provision of existing pediatric mental health services/resources for children with NDD across clinical settings, and local and online communities.

## 2. Methods

### 2.1. Search methods

Using a mixed-method triangulation approach, a multi-phase environmental scan was conducted (from May 2022 to July 2022). Two sources were used to conduct this scan: **Source 1**, across participating clinical settings in Quebec (Montreal, Laval, Laurentians) and **Source 2**, the local Montreal communities and web-based informational resources. Information found was organized and recorded on an online data extraction form searchable by section.

### 2.2. Inclusion and exclusion criteria

**Source 1** included all services from participating clinical settings that provided mental health support for children with disabilities. **Source 2** included pediatric mental health services in the local community and web-based informational resources for children with and without a disability. Private clinics were excluded in our scan to narrow the scope and primarily focus on the accessibility of low-cost pediatric mental health support. Only open-access web-based resources (in English and/or French) developed in North America were included. For the environmental scope of the scan, in-person services had to be offered across the participating jurisdiction areas (Montreal, Laval, Laurentians). All services/resources included needed a mental health component targeting children between 0 and 18 years of age. Given the multi-faceted nature of mental health supports, services/resources that had an identifiable aspect related to addressing children's emotional, psychological, and social well-being were included.

#### 2.2.1. Source 1: participating clinical sites

Following an outreach approach, clinical managers/coordinators of pediatric programs at **participating sites** were contacted to outline and describe the existing mental health care pathways and programs for children with NDD. We administered short online semi-structured interviews with participating clinical managers/coordinators using a pre-developed data extraction form (Supplementary Material).

#### 2.2.2. Source 2: local community services and online resources

A mapping synthesis approach was used to conduct a rapid online search of the available **local community services** and **online resources** for the mental health of children with and without NDD. Local community services included all pediatric mental health care services in Montreal, Laval, and the Laurentians. Web-based informational resources that provide mental health support for children with and without NDD were included. Findings for all services/resources were recorded on the data extraction form (Supplementary Material). The internet was searched to find local community services and online resources. Services/resources were called directly using the phone number provided online to find any missing information that could not be found during the online search.

### 2.3. Data collection

The data extraction form collected information describing mental health services/resources. Before extraction, services/resources were categorized based on their ***nature*** as *in-person*, *telehealth*, and/or *web-based informational*. Services were *in-person* when healthcare professionals provided face-to-face mental healthcare. *S*ervices were considered *telehealth* when provided by a mental health professional over video or the phone. *Web-based informational* resources were defined as online information that offers pediatric mental health support to the child, parents and/or family.

1) The ***general information*** section extracted details about each service/resource found, including the *director/clinical manager, phone number, website, email address, and location*. All information found was publicly available to the general population.
2) The ***method of initial access*** highlighted how the service/resource was accessed. We looked at if appointments were reserved over the *phone*, by *email*, or through an *online booking portal*. Services were considered *care by transfer* when patients were directly transferred from a healthcare service to mental health services through a clinician. Additionally, if the service offered was considered *immediate care* (e.g., walk-in clinic, emergency/crisis support, help phone line). Web-based informational resources were considered to have *no method necessary*.
3) The ***admission criteria*** category highlighted information about the participation condition(s). We identified if the services/resources were restricted for children with *specific disabilities*. We identified if the services/resources were restricted for children of a specific *age*. We identified if the service/resource had an additional *cost*. We identified if there was a *language* restriction by identifying what services/resources were offered only in English or French instead of being bi-lingual. Additionally, we reported which service/resource required a *referral* from another healthcare professional. We also recorded if the service/resource currently had a *waitlist*. Lastly, we identified if the patient's residential *geo-location* was an admission criterion, where only residents of a specific area could be admissible to receive the service/resource.
4) The ***target*** of the service/resource was defined as the person for whom the services were designed. This was broken down into the *child, parent* and/or *family*.
5) The ***focus/goal*** category was classified based on the specific aim/aims of the service/resource. *Assessment* services/resources evaluated and/or provided diagnostic information on the patient's mental health concerns. *Intervention* services/resources aimed to manage and treat mental health concerns. *Follow-up* services/resources (more specific to in-person and telehealth) provided patients with a post-intervention assessment to ensure the patient's mental health needs have been met. Services/resources were defined as *crisis/emergency* when they offered immediate support to mental health concerns (e.g., suicide attempts/ideations). *Redirection* services/resources referred patients to other mental healthcare services and/or offered information about other mental healthcare services.
6) The ***other*** section included any pertinent information about the service that did not “fit” one of the five categories. This section only included data collected from **Source 1**.

### 2.4. Data analysis

All three reviewers reviewed the data extraction form completed for each service/resource to ensure that data was not missing and was complete. All services/resources were divided and allocated based on whether they were in-person (including in-person with telehealth option), telehealth or web-based informational. Recorded information about the general information, admission/participation criteria, nature, and target of the service/resource was then inputted into a data form searchable by section. Extracted data for each section was inputted into the corresponding subsection in excel, and descriptive statistics were used to analyze the data. Additionally, a qualitative summary presented the “other” information reported by clinical managers at participating sites and was analyzed using a narrative analysis approach (27).

## 3. Results

**Figure 1:**
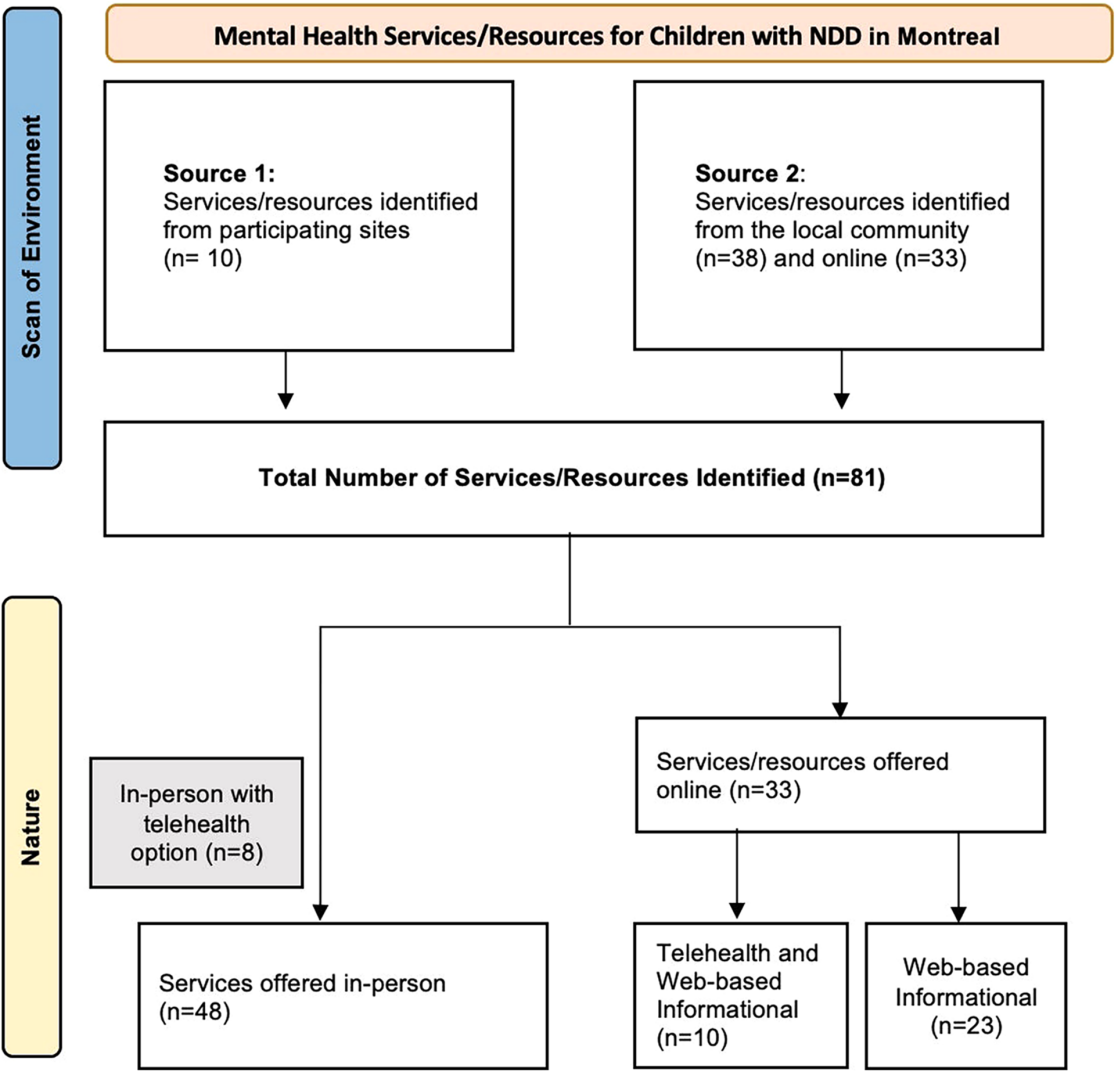
Flow diagram of mapped services/resources.

Three (*n* = 3) large urban pediatric rehabilitation sites and eight (*n* = 8) clinical managers and coordinators were interviewed for **Source 1**. The clinical managers identified ten programs (*n* = 10) that provided mental health support for children with NDD. For **Source 2**, thirty-eight (*n* = 38) services in the local community, ten (*n* = 10) telehealth services and twenty-three (*n* = 23) web-based informational resources were identified through the online search. The environmental scan included eighty-one (*n* = 81) services/resources (Figure 1). The services/resources were further categorized based on their nature, being in-person (*n* = 48), telehealth (*n* = 10) or web-based informational (*n* = 23). We noted that of the forty-eight (*n* = 48) in-person services identified, eight (*n* = 8) offered a hybrid model of care where telehealth was an available option. Table 1 presents the results from the scan described below.

**Table 1:** Results from the environmental scan.

Nature of resources	In-person, *n* = 48 (*n* = 8, telehealth option)	Telehealth, *n* = 10	Web-based Informational, *n* = 23	Total, *n* = 81
**General information, *n* (%)**				
Director/clinical manager	41 (85)	4 (40)	8 (35)	53 (65)
Phone number	48 (100)	6 (80)	15 (65)	69 (85)
Website	48 (100)	10 (100)	23 (100)	81 (100)
Email address	19 (40)	3 (30)	14 (61)	36 (44)
Location	48 (100)	10 (100)	10 (100)	81 (100)
**Method of initial care access, *n* (%)**				
Via phone	37 (77)	1 (10)	0 (0)	38 (47)
Via email	16 (33)	3 (30)	0 (0)	19 (23)
Via online booking portal	6 (13)	2 (20)	0 (0)	8 (10)
Care by transfer	11 (14)	0 (0)	0 (0)	11 (13)
Immediate care	1 (2)	6 (60)	0 (0)	7 (9)
No method necessary	0 (0)	0 (0)	23 (100)	23 (28)
**Admission criteria, *n* (%)**				
Disability specific	23 (47)	1 (10)	0 (0)	24 (29)
Restricted age	-	-	-	-
Age <11 (mean age 3.6)	8 (17)	0 (0)	N/A	8 (10)*
Other (mean age 14.5)	20 (42)	6 (60)	N/A	26 (33)*
Cost	2 (4)	0 (0)	0 (0)	2 (2)
Language restriction	5 (10)	1 (10)	3 (13)	9 (11)
Referral	32 (67)	0 (0)	N/A	32 (40)*
Waitlist	18 (38)	2 (20)	N/A	20 (24)*
Geo-location	21 (43)	0 (0)	0 (0)	21 (26)
**Target, *n* (%)**				
Child	48 (100)	10 (100)	15 (65)	69 (85)
Parent	34 (71)	4 (40)	18 (78)	56 (72)
Family	23 (47)	2 (20)	13 (56)	38 (46)
**Focus/goal, *n* (%)**				
Assessment	35 (73)	3 (30)	2 (9)	40 (49)
Intervention	46 (96)	9 (90)	6 (26)	61 (75)
Follow-up	13 (27)	0 (0)	0 (0)	13 (16)
Crisis/emergency	11 (23)	3 (30)	1 (4)	15 (19)

NDD, neurodevelopmental disabilities; *n*, number; %, percentage; N/A, not applicable.

* Based on the nature of web-based informational resources, the classification of having a referral or waitlist was not applicable. This may explain lower values found for total resources.

### 3.1. General information

Most general information about each service/resource was publicly available, including the director/clinical manager, phone number, website, and location (*n* = 53, 65%; *n* = 69, 85%; *n* = 81, 100%; *n* = 81, 100%, respectively). Fewer services/resources provided an email address (*n* = 36, 44%), which was seen most notably when looking at in-person resources (*n* = 19, 40%).

### 3.2. Method of initial access

**Figure 2:**
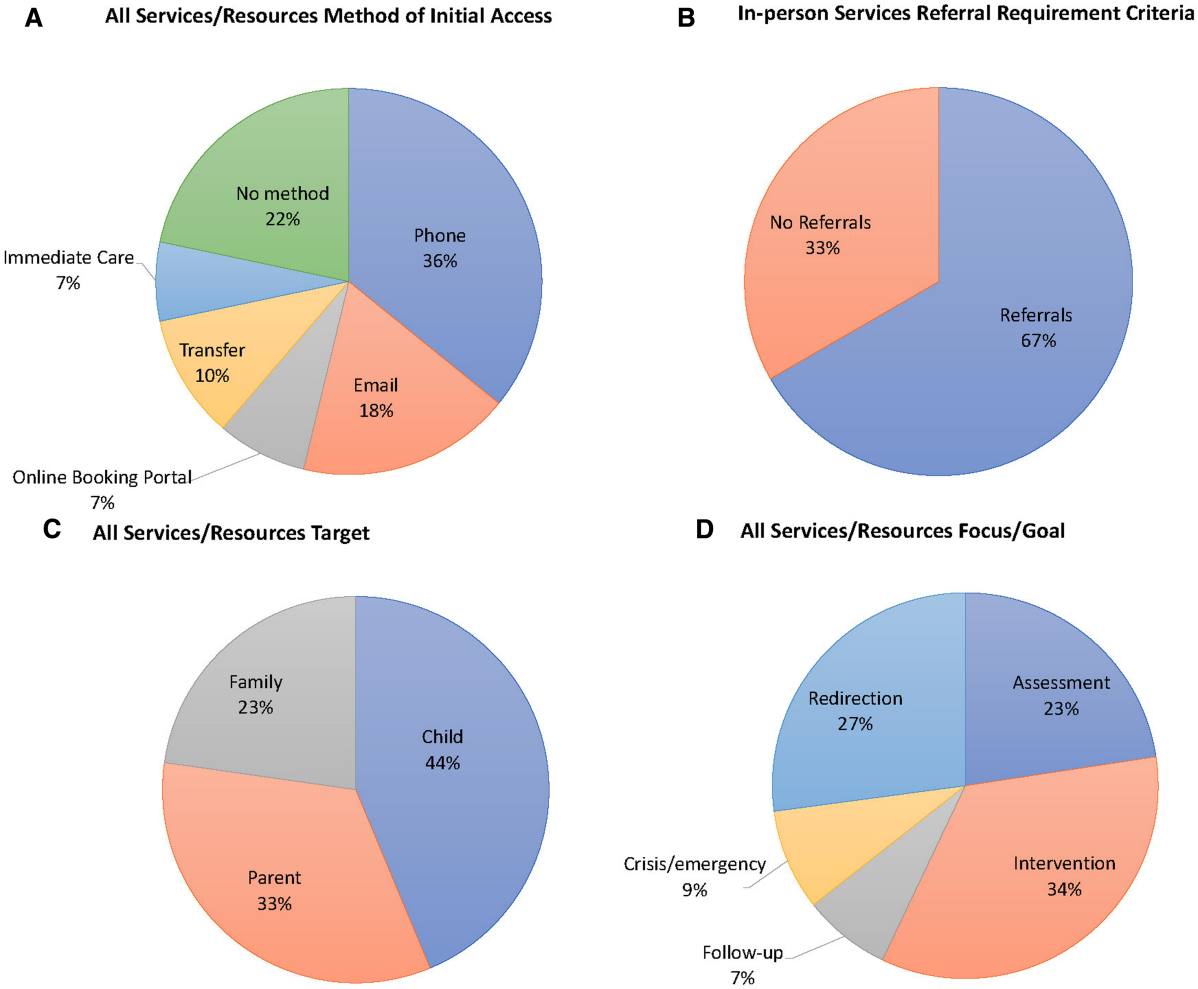
Pie chart representing the percentage of (**A**) the target of all services/resources; (**B**) the method of initial care access for all service/resources; (**C**) the target of all services/resources; (**D**) the focus/goal of all services/resources.

The primary method of care access in in-person services was through the phone (*n* = 37, 77%). Only a small percentage of in-person services had appointments reservable via email or an online booking portal (*n* = 16, 33%; *n* = 6, 13%, respectively). Eleven in-person services (*n* = 11, 14%) provided care by direct transfer from another health care service (e.g., emergency unit). Over half of the telehealth services offered immediate care through telecommunication (*n* = 6, 60%). Given the nature of web-based telehealth resources, there was no method of care coordination necessary (*n* = 23). Figure 2 highlights significant results about service/resource characteristics.

### 3.3. Admission criteria

**Figure 3:**
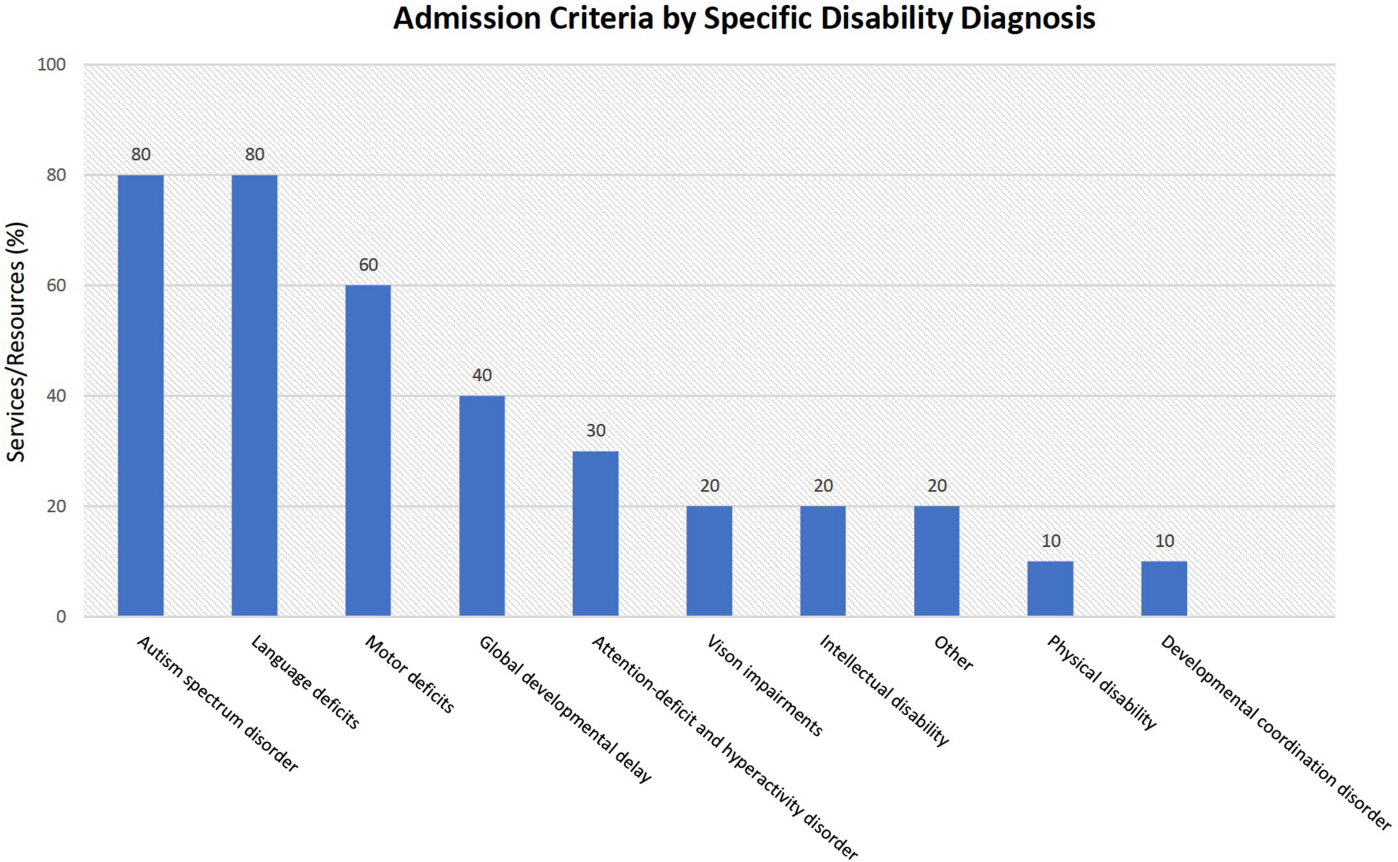
Admission criteria based on specific conditions.

Almost half of the in-person services (*n* = 23, 47%) had admission criteria specific for children with disabilities, including the type and/or severity [e.g. autism spectrum disorder (*n* = 8); language deficits (*n* = 8); motor deficits (*n* = 6); global developmental delay (*n* = 4); attention-deficit and hyperactivity disorder (*n* = 3); vision impairments (*n* = 2); intellectual disability (*n* = 2); other (*n* = 2); developmental coordination disorder (*n* = 1); physical disability (*n* = 1)] (Figure 3). Telehealth and web-based informational services/resources were tailored for all children, not specifically for children with disabilities.

In terms of the age requirements, twenty-eight (*n* = 28, 58%) of the in-person services and six (*n* = 6, 60%) of the telehealth services had specific age requirements that did not include all children ages 0–18 (e.g., children ages 5–11). Only eight (*n* = 8, 17%) in-person services were targeted for children under 11 years old (mean age = 3.6 years). No telehealth services offered care restricted to children under 11 years old. Twenty (*n* = 20, 42%) in-person services and six (*n* = 6, 60%) telehealth services were restricted by an older age range requirement (mean age = 14.5 years).

While only two (*n* = 2, 2%) services/resources had an additional cost, most were offered in both French and English (*n* = 72, 89%). Half of the in-person services (*n* = 32, 67%) required a referral from an external healthcare professional (e.g., registered psychologists and local community service centers). Referral from a healthcare professional was not required for telehealth and web-based informational services/resources (Figure 2). Additionally, 38% (*n* = 18) of in-person services had an identifiable waitlist. Nearly half of the in-person services (*n* = 21, 43%) had a geo-restriction causing limited access to the service (i.e., only residents of the area can access services).

### 3.4. Target

While all programs targeted the child experiencing mental health concerns, fewer in-person and telehealth services supported the parents’ mental health (*n* = 34, 71%; *n* = 4, 40%, respectively). Additionally, even fewer in-person and telehealth services targeted the mental health concerns of the entire family (*n* = 23, 47%; *n* = 2, 20%, respectively). We saw that a more significant number of web-based informational resources targeted the needs of parents and families (*n* = 18, 78%; *n* = 13, 56%, respectively). Results for all services/resources are shown in Figure 2.

### 3.5. Goal/focus

About half of services/resources focused on assessing mental health concerns (*n* = 40, 49%). Most services/resources focused on mental health intervention (*n* = 61, 75%). Only 16% of all services/resources (*n* = 13) incorporated follow-up support. Additionally, a few services/resources (*n* = 15, 19%) focused on mental health crises/emergencies. All web-based informational resources (*n* = 23) focused on redirection, providing information on local in-person mental health support. Results for all services/resources are shown in Figure 2.

### 3.6. Other

Clinical managers emphasized a lack of mental health care support and awareness for children with NDD. Additionally, they suggested that program funding for mental health support played a minor role but was not the causal factor. Clinical managers expressed that the most significant barrier was an insufficient number of pediatric mental health professionals to support patient demands. They also reported a sense of inadequate training among themselves and their staff when intervening with co-existing mental health demands of children with NDD. For instance, they convey that the intervention is often focused more on the behavioural, cognitive, and/or motor impacts of the child's disability instead of directly addressing emerging mental health concerns.

## 4. Discussion

The objective of this brief report was to explore and describe local existing pediatric mental health services/resources for children with NDD. Consolidating a map of existing services and resources into one categorized collection allowed us to highlight current gaps/optimization needs in services for children with NDD while identifying what populations are “falling through the cracks.” Additionally, we were able to indicate where future efforts are necessary to ascertain equal accessibility to services and benefits.

Only a limited number of services utilize telehealth methods to intervene and assess pediatric mental health concerns. Telerehabilitation is an emerging and promising approach for children with NDD when face-to-face care is limited (28). Given the uncertainties of the COVID-19 pandemic and the increased risk for health concerns of children living with NDD, having a hybrid model of service delivery (in-person and telehealth) may support the mental healthcare needs of children living with NDD (29). A recent systematic review on telerehabilitation for children with disabilities has shown the effectiveness of telerehabilitation and its equivalency to in-person care (28). Telehealth is also convenient and cost-effective, with no geo-location barriers (30). Additionally, telehealth favors high-risk populations, such as children with disabilities, who often have underlying health conditions and need to receive care safely at home (30). Recent studies have also shown that telerehabilitation can promote family-centeredness during intervention/treatment, facilitating care delivery in the comfort of the child's home environment and with family support nearby (31). The complementarity of including a hybrid approach would ensure that the care is tailored to what works best for the family, considering their barriers and the effectiveness of the care. Thus, the addition of telehealth as a mode of care alongside face-to-face mental health services promotes patient and caregiver autonomy and may improve how care is received.

We found that the primary method of initial care access for pediatric mental health services was done through the phone. Traditionally, a patient's first medical appointment is made with schedulers over the telephone (32). These methods are based on verbal communication with real people and allow maximum flexibility in complicated situations (32). However, coordinating care over the phone may be timely as the appointment is limited by the availability of appointments and the schedulers on the phone line (33). Patient or caregiver satisfaction with appointment booking is influenced by their ability to book at the right time with the right health service providers (34). Several studies have conducted satisfaction surveys and found that web-based appointment scheduling is an essential feature that allows for a faster appointment time and encourages patients and caregivers to use the health service again (33, 35). Having a web-based appointment scheduling portal for in-person mental health services could reduce the time to intervention, thus supporting timely access to pediatric mental health care.

We determined that access to mental health support can be complicated, as almost all in-person services have strict admission criteria, including diagnosis, a specific age requirement, a referral process, a waitlist, and a restricted geo-location. Notably, half of the services identified were specialized, providing targeted support based on the child's condition. We found that most services were tailored for conditions such as autism spectrum disorder, language deficits and motor deficits. However, we found only one service tailored for children with physical disabilities and none for children with diagnosed cerebral palsy. Children with physical disabilities such as cerebral palsy are particularly vulnerable to mental health problems due to the associated motor and physical complications that intensify these challenges (36). Given that cerebral palsy is the most common physical childhood disability, specific services must be available to support this population's mental health needs (37). We found that some services had an age requirement, which could have improved the specialization of mental health care as the child's age and development of NDD are likely to be considered. However, few services are available for younger children (e.g., under 11 years old), suggesting that mental health concerns in the younger clientele may be neglected. Children with untreated mental health concerns are known to overutilize health services leading to an increased prevalence of long-term mental health conditions and increased healthcare costs (38). Thus, if mental health concerns present early, assessment and treatment services must be available and easily accessible (39). Additionally, many mental health services require a referral assessment with another healthcare professional, which often causes unnecessary wait times for children to receive the mental health care they need. Timely access to services is critical when providing mental health care for children with NDD, given that extended wait time may intensify mental health concerns (40). Moreover, less than one-third of all services/resources were specialized to cater to the mental health needs of children with NDD. It is found that children with NDD experience increased difficulty accessing specialty care due to limited clinician resources and geographic distribution of specialists (41, 42). Given the increased prevalence of complex mental health needs in children with NDD, there is a need to increase the available specialized services to support these children. However, it is ever more challenging to meet the specialized demand levels for children with NDD due to lengthy wait times for all pediatric mental health services.

We determined that most services/resources targeted the child's mental health concerns while including additional support for the parent's mental well-being. Fewer resources provided support targeting the family, where the care is directly focused on caregivers and their children together. Family-centred care has been established as a best practice model for child disability services internationally (43). Given the strong interlink between the child's and caregiver's health and well-being, including the family in care is essential (44, 45). One study showed that including parental support along with individual child-targeted mental health interventions had an 80% benefit in participating families (46). In line with the evidence, mental health professionals should consider using a family-centred approach when providing mental health support for children with NDD.

In-person services mainly focused on assessment and intervention for pediatric mental health concerns. In contrast, telehealth services primarily focused on crisis/emergency support (e.g., suicide helplines). Most web-based informational resources provided redirection support with information about local mental health clinics or other reputable online resources. Telehealth has shown to be an effective healthcare assessment and intervention method for many NDDs and has proven to be well-accepted by parents and healthcare providers (47, 48). Given patient differences in the method of treatment effectiveness, professionals should use an individualized approach to assess if telerehabilitation is the best fit for their patient with an NDD (28). Overall, increasing the use of telehealth could support the demand for accessible and timely pediatric mental health care for children with NDD. Limited services/resources incorporated a follow-up component. When mental health concerns are critical, follow-up care could be essential to reduce hospital readmissions and other adverse healthcare costs (49).

Given the dense population of approximately 4.3 million in metropolitan Montreal, a relatively small number of pediatric mental health resources were found (50). However, this could be explained given the explored nature of only public resources and the exclusion of resources in the private healthcare sector. Additionally, clinical managers from participating sites expressed the need for more public pediatric mental health care professionals to support the demand. Evidence shows a large service gap for children with mental health conditions, which is arguably even more prominent than the widely recognized gap for adults (51). In one study, primary care practitioners expressed how limited providers for mental health services are the most highly endorsed barrier (52). Clinical managers also expressed that a lack of awareness and knowledge about mental health concerns and prevalence in children with NDD may also play a role in the quality of mental health support. A scoping review exploring the barriers and enablers to accessing mental health in children with intellectual disabilities shows that lack of clinician training acts as a barrier to accurate diagnosis and care (53). Additionally, a “diagnostic overshadowing” phenomenon may occur when healthcare professionals misclassify symptoms as an expression of the existing disability rather than a mental health condition (54). Thus, increasing the limited coordination of knowledge between the disability and mental health service sectors could increase the effectiveness of resources for individuals with NDD.

Many unmet needs have been identified for children living with NDD and mental health concerns. Initiatives are needed to improve service accessibility, inclusive care, longitudinal follow-up, and patient and family-centred care to support mental health outcomes for children with NDD.

## 5. Limitations

A few limitations can be identified following an environmental scan methodology. The first is that the search strategy used is less rigorous than other research methods. Given our aim to get a snapshot of local mental health care rather than an exhaustive search, only “Grey literature” was scanned. Therefore, given the unstructured scope of the scan, we may have missed specific, less visible services/resources and characteristics that could have been relevant to our project. Another methodological consideration concerns findings, as the interpretation and identification of broad themes often need to be more comprehensive (55). Additionally, **Source 1** of the scan is from the perspective of healthcare providers and may not necessarily align with patient and caregiver perceptions. Future research may consider mapping services/resources from the caregivers’ perspective. Lastly, the data collected for waitlist prevalence may be a limitation as we needed to determine the waitlist existence or duration for some services. Therefore, the waitlist results should be generalized cautiously.

## 6. Implications for research and practice

This brief report aimed to describe existing pediatric mental health services/care pathways for children with NDD across participating clinical settings, local communities, and online resources/support. Through doing so, we consolidated existing services and resources for pediatric mental health into one collection. We indicated common themes to understand where future efforts are necessary to ascertain equal accessibility to services and benefits for children with NDD. The findings of this scan show the importance of providing specialized and multifaceted mental health care for children with NDD and their families. Future efforts to create an online platform searchable by mental health service/resource characteristics could improve tailored and effective mental health care accessibility. Although we were able to identify gaps in existing mental health management, more research is needed to further understand the reality from the perspective of clinicians and caregivers of children with disabilities. Through doing so, we will be able to appreciate existing similarities and differences to identify improvements needed in clinical practice and models of care.

## Data Availability

The raw data supporting the conclusions of this article will be made available by the authors, without undue reservation.
