# Supplementary material for: Mental health services and resources for children with developmental disabilities and their families: scan of local practices, gaps, and opportunities created
Source: Front Rehabil Sci. 2023 May 30;4:1118769. doi: 10.3389/fresc.2023.1118769 (PMC10267822; doi:10.3389/fresc.2023.1118769)
Supplement: Supplementary file 1 [file Datasheet1.pdf]

Nature of Service/Resource (Select one of more that apply):

☐ In-person ☐ Telehealth ☐ Web-based informational

Section 1:

What is general information about the services/resources?	
Name of service/resource	
Name of director and/or clinical coordinator	
Title (Dr., etc.)	
Phone number	
Email Address	
Location	
Website	

Section 2*:*

What is the services/resources method of initial access?	***Select one or more that apply***	***Comments***
Are appointments reserved over the phone?	☐	
Are appointments reserved over email?	☐	

Are appointments reserved on an online booking portal?	☐	
Are services care by transfer (e.g., direct transfer from healthcare service to mental healthcare)?	☐	
Was there no method necessary (e.g., web-based informational)?	☐	

Section 3:

What are the admission/participation criteria for the service/resource?	***Select one or more that apply***	***Comments***
Specific Disability, *If yes, specify what disability*	☐	
Age of the child (Years)	☐	
Cost	☐	
Language (e.g., only in English or French), *If yes, specify what language*	☐	
Referral	☐	
Wait-list	☐	
Geo-location	☐	

Section 4:

Who does the service/resource target? *(Does the program address the needs of the child, the parent, or both? If both specify details for each group and if there are any differences).*	***Select one or more that apply***	***Comments***
Child	☐	
Parent	☐	
Both (Family)	☐	

Section 5:

What is the goal/focus of the service/resource?	***Select one or more that apply***	***Comments***
Assessment	☐	
Intervention	☐	
Follow-up	☐	
Crisis/emergency management	☐	
Redirection	☐	
Other	☐	

Section 6:

Any other pertinent information that you would like to share with us about this program:
